# Ernst Rüdin and the State of Science

**DOI:** 10.1371/journal.pgen.1005707

**Published:** 2015-11-18

**Authors:** Michael Yudell

**Affiliations:** Department of Community Health & Prevention, Drexel University School of Public Health, Philadelphia, Pennsylvania United States of America; Seattle Children's Research Institute, UNITED STATES

It should be no surprise to the readers of this journal that the intersection of eugenics, psychiatry, and National Socialism in the work of Swiss-born German scientist and physician Ernst Rüdin ended poorly for the victims of his abominable ideas. As one of the primary architects of Nazi eugenics, Rüdin’s impact was felt most immediately in the euthanasia programs that his ideas instigated and he helped design as chair of the Committee for Racial Hygiene and Racial Policy at the Ministry of the Interior in Hitler’s Germany. That committee gave rise to the “Law for the Prevention of Hereditarily Diseased Offspring,” and that law led to between 350,000 and 400,000 German sterilizations during the Nazi rule [1–2].

But as Kösters et al. [1] illustrate in their important paper in this issue of *PLOS Genetics*, there is more to Rüdin’s story than just his role in and impact on Nazi eugenic programs. Their paper offers new insights into long-standing debates about how to remember Rüdin and what his story can tell us. Foremost, their paper draws our attention to a dark chapter in the history of genetics and examines Rüdin’s role therein. Of particular interest is the way Rüdin, generally credited as the founder of psychiatric genetics, was deeply entangled in eugenic thought and practice and how his eugenic thinking was inseparable from his genetic research. In that research, Rüdin worked to develop methodologies integrating emerging statistical methods with Mendelian genetics to predict how mental illness passed within families. His goal: an “empirical hereditary diagnosis” of mental illness.

But Kösters et al. show how eugenics not only shaped Rüdin’s research questions, but shaped the very fabric of his research endeavor. As their paper reveals, fundamental to Rüdin’s work was “to provide scientific evidence that severe mental illness was inherited, thus strengthening the case of eugenic measures.” Rüdin hoped to use genetics in the service of what he ominously called “therapeutic reform.”

Second, by showing that Rüdin suppressed his own findings regarding the inheritance of “manic-depressive insanity” because they offered contradictory evidence to his eugenic worldview, Kösters et al. draw our attention to how far Rüdin was willing to let his ideology drive his science and suppress negative findings to that end. The example provided is a study conducted in the early to mid-1920s (even before the rise of the Third Reich but as eugenic ideas were growing in strength in Germany) on the “Inheritance of Manic-Depressive Insanity” that one biographer describes as “probably the most significant of Rüdin’s work.” However, the results from this study did not conform to Rüdin’s worldview, and did not support his idea that he could use hereditary data to predict “the probability that a particular individual would develop an illness.”

Rüdin would never publish these findings. Why not? After all, the “Inheritance of Manic-Depressive Insanity” was a completed manuscript ready for publication. Kösters et al. believe that Rüdin’s “demands for negative eugenic measures against patients with affective disorders and their families could not be justified on the grounds of the hereditary figures he had calculated” in the unpublished manuscript. Thus, the paper and its important findings disappeared, and Rüdin and his acolytes in both Europe and the United States continued to search for genetic justifications for their eugenic ideas. To be clear, it is not as if had Rüdin published these negative findings would his own ideology or the trajectory of Nazi eugenics have been altered. Indeed, the findings could have been justified, as Rüdin did in the unpublished manuscript, by citing the “preliminarity” of the findings and the need for larger sample sizes in the hope to find the desired effect.

So what does the Rüdin story tell us?

Well, for one thing, as Kösters et al. argue, it turns out that Rüdin’s calculations in this unpublished work have largely stood the test of time despite more than seventy years of theoretical and technological advances in genetics. “The search for replicable gene variants leading to the onset of affective disorders continues,” Kösters et al. tell us.

But Rüdin’s suppression of his own findings also raises important ethical issues that persist today concerning both what science loses when negative findings are unpublished, as well as ethical questions about the social character of science.

For example, Kösters et al. cite the work of geneticist Peter Propping, who considers the failure to report negative findings “a silent coalition… between an author and an editor.” Publication bias in both basic science and clinical research can have far reaching consequences, including the failure to report negative findings, thus skewing the evaluation and approval process of new drugs. Publication bias can also lead to a fruitless pursuit of research that has already been completed, and calls into question the overall integrity of research [3–5]. One recent study suggests that more a quarter of all clinical trials remain unpublished [6].

It is of course tempting to turn away from the story of Rüdin—a eugenicist and Nazi—and disregard the lessons here. Nonetheless, Rüdin provides a powerful example of the scientists’ capacity to do wrong (selective non-publication of results) and to do great harm (the impact of his eugenic theories on Nazi policy). His example serves as a reminder that our sociology can impact our science in ways, both big and small. For genetics, that includes the way a variety of forces, including availability of research funds, the pressures of obtaining tenure, or an embrace of genetic reductionism can shape or limit research questions and study design.

Rüdin likely never considered the way his embrace of eugenics and National Socialism, as Kösters et al. point out, compromised “the scientific quality of his empirical studies.” But we, as natural and social scientists, should be ever vigilant in considering the biases and influences of social and political context on our own work. Rüdin’s story reminds us that the risks of not doing so are a persistent challenge to the ethical practice of science.
